# Establishment and Application of a Triplex Real-Time RT-PCR Assay for Differentiation of PEDV, PoRV, and PDCoV

**DOI:** 10.3390/v15061238

**Published:** 2023-05-25

**Authors:** Wenwen Hou, Maodi Fan, Zhenbang Zhu, Xiangdong Li

**Affiliations:** 1 Jiangsu Co-Innovation Center for Prevention and Control of Important Animal Infectious Diseases and Zoonoses, College of Veterinary Medicine, Yangzhou University, Yangzhou 225009, China; 2 Key Laboratory of Protection & Utilization of Biological Resources in Tarim Basin, College of Life Sciences, Tarim University, Alar 843399, China

**Keywords:** triplex real-time RT-PCR, porcine epidemic diarrhea virus, porcine rotavirus, porcine deltacoronavirus

## Abstract

Porcine viral diarrhea is very common in clinical practice and has caused huge losses to the pig industry. Porcine epidemic diarrhea virus (PEDV), porcine rotavirus (PoRV), and porcine deltacoronavirus (PDCoV) are important pathogens of porcine viral diarrhea. Co-infection situations among these three viruses in clinics are common, which increases the difficulty of differential diagnosis. Currently, polymerase chain reaction (PCR) is commonly used to detect pathogens. TaqMan real-time PCR is more sensitive than conventional PCR and has better specificity and accuracy. In this study, a triplex real-time RT-PCR assay based on TaqMan probes was developed for differential detection of PEDV, PoRV, and PDCoV. The triplex real-time RT-PCR assay developed in this study could not detect unrelated pathogens and showed satisfactory specificity, sensitivity, repeatability, and reproducibility with a limit of detection (LOD) of 6.0 × 10^1^ copies/μL. Sixteen clinical samples were used to compare the results of the commercial RT-PCR kit and the triplex RT-PCR for PEDV, PoRV, and PDCoV detection, and the results were completely consistent. A total of 112 piglet diarrhea samples collected from Jiangsu province were next used to study the local prevalence of PEDV, PoRV, and PDCoV. The positive rates of PEDV, PoRV, and PDCoV detected by the triplex real-time RT-PCR were 51.79% (58/112), 59.82% (67/112), and 2.68% (3/112), respectively. The co-infections of PEDV and PoRV were frequent (26/112, 23.21%), followed by the co-infections of PDCoV and PoRV (2/112, 1.79%). This study established a useful tool for simultaneous differentiation of PEDV, PoRV, and PDCoV in practice and provided valuable information on the prevalence of these diarrhea viral pathogens in Jiangsu province.

## 1. Introduction

Porcine viral diarrhea leads to a high mortality rate in piglets and causes serious economic losses to the pig industry all over the world [1]. Porcine epidemic diarrhea virus (PEDV), porcine rotavirus (PoRV), and porcine deltacoronavirus (PDCoV) are important clinical pathogens causing viral diarrhea in pigs [2,3]. PEDV is a member of the genus *Alphacoronavirus* in the family *Coronaviridae*, which is an enveloped, single-stranded positive-sense RNA virus. PEDV particles are spherical with different sizes; the average diameter is about 130 nm, and the total length of the PEDV genome is about 28 kb [4]. Porcine epidemic diarrhea (PED) was first reported in England in 1971 [5], then broke out again in Belgium in 1977 [6]. The first report of PED in China was in 1973, and it was not confirmed as PED until 1984 [7]. Since October 2010, PEDV has suddenly erupted on a large scale in China, and the mortality of newborn piglets has increased significantly, which indicates the emergence of a new variant virulent strain. In April 2013, the United States of America experienced the first outbreak of highly pathogenic PEDV infection [8], and the epidemic has gradually spread to a wide range. Currently, PEDV has spread widely to many parts of the world [7]. PoRV belongs to the genus *Rotavirus* in the family *Reoviridae* [9]. PoRV is a non-enveloped, double-stranded RNA virus [10]. The full length of the PoRV genome is about 18,500 bp, which is composed of 11 segments of dsRNA. They encode six structural proteins (VP1~VP4, VP6, and VP7) and five non-structural proteins (NSP1~NSP5/6). PoRV is divided into seven serogroups (A–J) based on VP6 [11]. Subgroup A rotavirus is the main rotavirus causing gastrointestinal disease in swine, with high pathogenicity and prevalence. Subgroup B was first reported and described as a rotavirus-like agent in 1985 [12]. Subgroup C was first isolated from swine in 1980. Currently, subgroups A, B, C, E, and H have been described in swine. Due to its complex co-infection situation, high variability, and worldwide distribution, PoRV has gradually become an important porcine diarrhea pathogen [11]. PDCoV is a member of the genus *Deltacoronavirus* in the family *Coronaviridae*. PDCoV is an enveloped, single-stranded positive-stranded RNA virus, and its genome size is approximately 25.4 kb (excluding the poly A-tail) [13]. In 2012, PDCoV was first reported in Hong Kong, China [14], then it was reported in the United States of America in 2014 and quickly spread to many states in a very short time [15,16,17]. The virus was also detected in Canada, South Korea, and Thailand in succession [18].

PEDV, PoRV, and PDCoV can cause similar clinical symptoms, including vomiting, diarrhea, and dehydration in piglets. Meanwhile, the pathological changes among them are also very similar and difficult to distinguish. In addition, co-infections and secondary infections among PEDV, PoRV, and PDCoV are very common, which makes clinical diagnosis quite difficult [19]. Therefore, there is an urgent need in the clinic to establish a rapid and efficient molecular method to differentiate them.

## 2. Materials and Methods

### 2.1. Viruses, Primers, and Probes

The DNA samples of pseudorabies virus (PRV), porcine circovirus 2 (PCV2), porcine circovirus 3 (PCV3), and cDNA samples of PEDV, PoRV, PDCoV, classical swine fever virus (CSFV), and porcine reproductive and respiratory syndrome virus (PRRSV) were stored in our laboratory at −20 °C until use. To acquire specific primers and probes, the primers and probes of PDCoV were adapted from the reference [20]. For PEDV and PoRV, we downloaded at least 20 genome sequences of PEDV and PoRV from NCBI for comparison and analysis. Primer Express 3.0 software (Applied Biosystems, Foster City, CA, USA) was used to design the primers and probes based on their most conserved regions. The TaqMan probes for PEDV, PoRV, and PDCoV were labeled with FAM, ROX, and TAMRA at the 5′ end, respectively, and all quenchers at the 3′ end were MGB (Table 1). Primers and probes were synthesized in Sangon (Shanghai, China).

### 2.2. Clinical Samples and Nucleic Acid Extraction

A total of 128 small intestine tissue samples from piglets with diarrhea symptoms were collected from 16 pig farms in 10 cities in Jiangsu province from 2021 to 2022. Among them, 16 samples were used for comparison between commercial single-plex real-time RT-PCR detection kits (Beijing Anheal Laboratories Co., Ltd.) and the established triplex real-time RT-PCR assay in this study. The rest of the 112 samples were only used to investigate the prevalence of PEDV, PoRV, and PDCoV using the triplex real-time RT-PCR assay. The mixture of intestinal contents and PBS was in a 1:5 ratio and subjected to centrifugation at 4 °C, 5000 rpm, for 10 min. The supernatant was then used to extract RNA using TRNzol Universal Reagent (DP424) (TIANGEN BIOTECH, Beijing, China) and reverse transcribe into first-strand cDNA using HiScript III 1st Strand cDNA Synthesis Kit (+gDNA wiper) (Vazyme, Nanjing, China). The steps of reverse transcription included denaturation of the RNA template, removal of genomic DNA, and synthesis of the 1st strand cDNAs. A total volume of 20 μL of reaction solution was instantly centrifuged and placed in an applied biosystems PCR cycler (Thermo Fisher Scientific, Waltham, MA USA). After 25 °C for 5 min, 37 °C for 45 min, and 85 °C for 5 s, the synthesized cDNA was used as a template for triplex real-time RT-PCR or commercial single-plex real-time RT-PCR.

### 2.3. Development and Optimization of the Triplex Real-Time RT-PCR

The concentrations of primers and probes were optimized as previously described [21]. After optimization, the triplex real-time RT-PCR reaction in a total volume of 20 μL was as follows: Premix Ex Taq 10 μL (Takara, China), cDNA templates 2 μL, primers (PEDV-M-F/R, PoRV-NSP5-F/R, or PDCoV-N-F/R) (10 μM) 0.5 μL for each of them, probes (PEDV-M-P, PoRV-NSP5-P, or PDCoV-N-P) (10 μM) 0.5 μL for each of them, and ddH_2_O 3.5 μL. The triplex real-time RT-PCR amplification was performed on the applied biosystems QuantStudio3 (Thermo Fisher Scientific, USA); the amplification condition was set at 95 °C for 30 s, followed by 40 cycles of 95 °C for 5 s and 60 °C for 30 s, and the fluorescent signal was detected at the end of the extension step in each cycle.

### 2.4. Standard Curve Generation of the Triplex Real-Time RT-PCR

Amplified fragments with PEDV-M-F/R, PoRV-NSP5-F/R, or PDCoV-N-F/R were synthesized and cloned into the pUC57 vector by Genewiz (Suzhou, China). The plasmid was then used as the standard positive control. The concentration of the plasmid was converted to copy number using the following formula: y (copies/μL) = (6.02 × 10^23^) × (x(ng/μL) × 10^−9^ DNA)/(DNA length × 660) [22]. The 10-fold serially diluted standard plasmids (6.0 × 10^1^–6.0 × 10^6^ copies/μL) were used as templates to generate the standard curve of the triplex real-time RT-PCR assay.

### 2.5. Specificity and Sensitivity of the Triplex Real-Time RT-PCR

To evaluate the specificity of the established triplex real-time RT-PCR assay, DNA samples of PRV, PCV2, PCV3, and cDNA samples of CSFV and PRRSV were applied. The sensitivity and limit of detection (LOD) of the triplex real-time RT-PCR assay developed in this study were verified by using 10-fold serially diluted standard plasmids (6.0 × 10^0^–6.0 × 10^6^ copies/μL).

### 2.6. Repeatability and Reproducibility of the Triplex Real-Time RT-PCR

To assess the repeatability and reproducibility of the triplex real-time RT-PCR assay, three different concentrations of 10-fold serially diluted plasmid (6.0 × 10^6^, 6.0 × 10^4^, and 6.0 × 10^2^ copies/μL) were used to test the intra-assay variability and the inter-assay variability. For intra-assay variability, the assay was repeated three times for each dilution on the same day. As for inter-assay variability, each dilution was tested in six independent experiments by two operators on different days according to MIQE guidelines [23]. The coefficients of variation (CVs) of Ct values were calculated from the intra-assay and inter-assay results.

### 2.7. Comparison of the Triplex Real-Time RT-PCR with the Commercial Single-Plex RT-PCR Kit

Sixteen piglet diarrhea samples collected from Jiangsu Province that have been tested positive for PEDV, PoRV, or PDCoV by a commercial single-plex real-time RT-PCR detection kit were used to verify the established triplex real-time RT-PCR assay. The sixteen samples were tested for single infections or co-infections with PEDV, PoRV, or PDCoV.

### 2.8. Clinical Application of the Triplex Real-Time RT-PCR

A total of 112 small intestine tissue samples of piglets with diarrhea symptoms collected from 16 pig farms in 10 cities of Jiangsu province from 2021 to 2022 were used to study the prevalence of PEDV, PoRV, and PDCoV using the established triplex RT-PCR.

### 2.9. Sequencing and Phylogenetic Analysis

Representative virus strains from the positive pig farms were selected for Sanger sequencing and phylogenetic analysis (seven PEDV strains, eight PoRV strains, and one PDCoV strain). The S genes of PEDV and PDCoV and the VP4 and VP7 genes of PoRV were amplified by conventional RT-PCR. The amplicons of PEDV and PDCoV were used for sequencing, and the VP4 and VP7 genes of PoRV amplicons were linked with the pMD-19T vector before sequencing. Sequence alignments and phylogenetic trees were performed by MEGA 11 (Mega Limited, Auckland, New Zealand).

## 3. Results

### 3.1. Primers and Probes Design and Concentration Optimization

The forward primer, reverse primer, and probe of PDCoV targeted the N gene and were adapted from the reference [19]. For PEDV and PoRV, genome sequences of at least 20 reference strains were downloaded from the NCBI GenBank database for comparison and analysis. The most conserved gene sequences in the M gene of PEDV and the NSP5 gene of PoRV were selected for the design of primers and probes. The 5′ ends of the probes used in this study were labeled with different fluorophores to ensure that there was no interference among the fluorescent signals of different viruses. The sequences of primers and probes were listed in Table 1. The concentrations of primers and probes were optimized as described in the Materials and Methods section. The same reaction parameters were used throughout the study.

### 3.2. Standard Curve of the Triplex Real-Time RT-PCR

**Figure 1:**
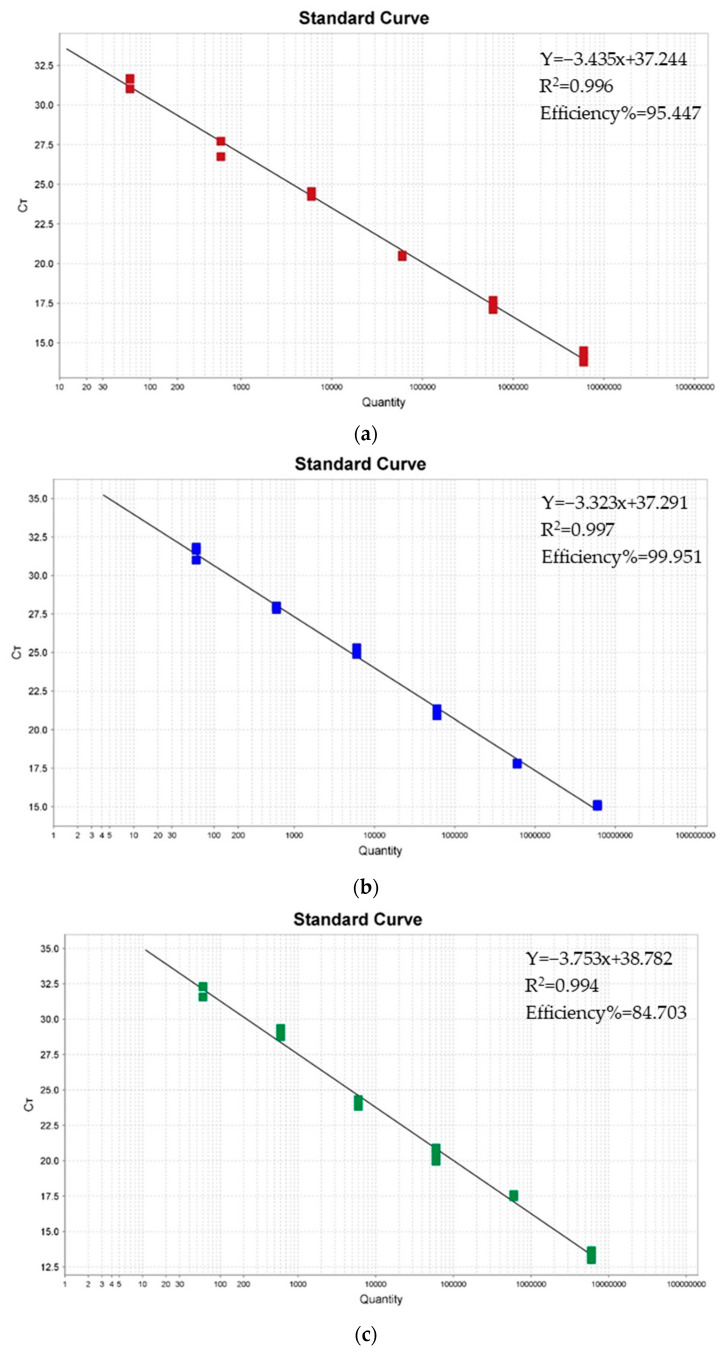
Standard curves of the triplex real-time RT-PCR assay. (**a**) The standard curve for porcine epidemic diarrhea virus (PEDV); (**b**) the standard curve for porcine rotavirus (PoRV); (**c**) the standard curve for porcine deltacoronavirus (PDCoV).

As shown in Figure 1, the standard curves of the triplex real-time RT-PCR assay were generated by using 10-fold serially diluted standard plasmids, ranging from 6.0 × 10^1^ to 6.0 × 10^6^ copies/μL. The slopes of the standard curve for PEDV, PoRV, and PDCoV were −3.435, −3.323, and −3.753, respectively. The correlation coefficients R^2^ for PEDV, PoRV, and PDCoV were 0.996, 0.997, and 0.994, respectively.

### 3.3. Standard Curve of the Triplex Real-Time RT-PCR

**Figure 2:**
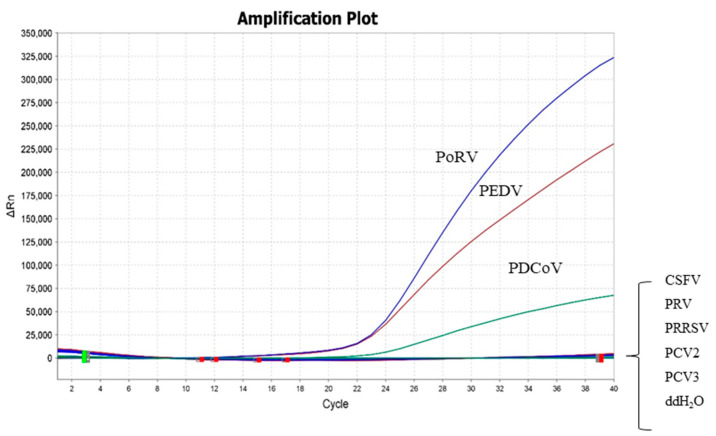
Specificity test of the triplex real-time RT-PCR assay. FAM, ROX, and TAMRA fluorescent signals were monitored by the triplex real-time RT-PCR assay, and the 6.0 × 10^4^ copies/μL of 10-fold serially diluted standard plasmid was used as a positive control. No fluorescent signal was observed for cDNA or DNA samples of other viruses.

The 6.0 × 10^4^ copies/μL of 10-fold serially diluted standard plasmid was used as a positive control, and ddH_2_O was used as a negative control. DNA samples of other common porcine viruses, including PRV, PCV2, PCV3, and cDNA samples of CSFV and PRRSV, were used to evaluate the specificity. The amplification curves showed that only corresponding FAM, ROX, and TAMRA signals for PEDV, PoRV, and PDCoV could be specifically detected, while no fluorescence signal was detected for other viruses (Figure 2). Furthermore, the fluorescent signals of these three viruses did not interfere with each other. The above results indicated that the established assay had high specificity.

**Figure 3:**
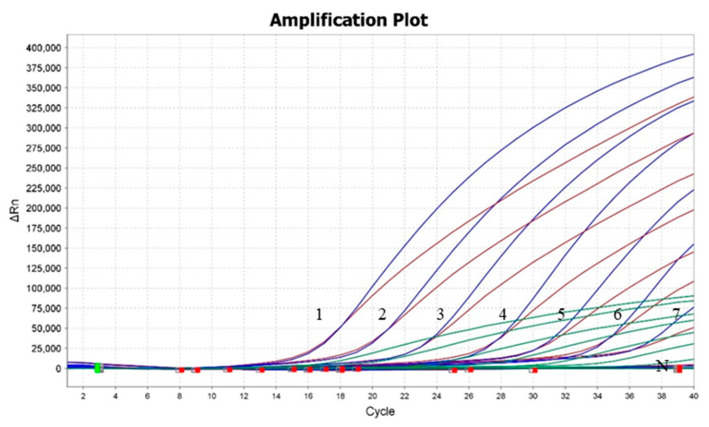
Sensitivity test of the triplex real-time RT-PCR assay. Labels 1–7 indicated the different concentrations of plasmids (6.0 × 10^6^, 6.0 × 10^5^, 6.0 × 10^4^, 6.0 × 10^3^, 6.0 × 10^2^, 6.0 × 10^1^, and 6.0 × 10^0^ copies/μL, respectively). The detection of PEDV, PoRV, and PDCoV was shown by red lines, blue lines, and green lines, respectively.

The sensitivity of the triplex real-time RT-PCR assay was determined by using the 10-fold serially diluted standard plasmids (6.0 × 10^0^–6.0 × 10^6^ copies/μL). ddH_2_O was used as a negative control. Our results showed that the LOD of the triplex real-time RT-PCR assay for detecting PEDV, PoRV, and PDCoV was 6.0 × 10^1^ copies/μL (Figure 3).

### 3.4. Repeatability and Reproducibility of the Triplex Real-Time RT-PCR

The intra-assay variability (repeatability) and inter-assay variability (reproducibility) were tested using three different concentrations of 10-fold serially diluted plasmid (6.0 × 10^2^, 6.0 × 10^4^, and 6.0 × 10^6^ copies/μL), and the results showed that the intra-assay and inter-assay CVs of Ct values ranged from 0.35% to 4.76% and 0.74% to 4.34%, respectively (Table 2). The results indicated that the triplex real-time RT-PCR assay established in this study had satisfactory repeatability and reproducibility.

### 3.5. Comparison of the Triplex Real-Time RT-PCR with the Commercial Single-Plex Commercial RT-PCR Kit

Sixteen clinical samples were used to compare the results of the commercial RT-PCR kit and the triplex RT-PCR for PEDV, PoRV, and PDCoV detection. These 16 samples had been confirmed as PEDV, PoRV, or PDCoV positive using a commercial single-plex real-time RT-PCR detection kit. As shown in Table 3, the results shown in the commercial real-time PCR detection kit and triplex real-time RT-PCR assay were completely consistent.

### 3.6. Clinical Application of the Triplex Real-Time RT-PCR

Furthermore, a total of 112 piglet diarrhea samples collected from Jiangsu province were used to investigate the local prevalence of PEDV, PoRV, and PDCoV. The positive rates of PEDV, PoRV, and PDCoV detected by the triplex real-time RT-PCR assay were 51.79% (58/112), 59.82% (67/112), and 2.68% (3/112), respectively. Furthermore, 26 out of 112 samples (23.21%) were found to be co-infected with PEDV and PoRV, and 2 out of 112 samples (1.79%) were found to be co-infected with PDCoV and PoRV (Table 4). More specific information about the detection results of 112 clinical diarrhea samples is shown in Table S1.

### 3.7. Gene Sequencing and Phylogenetic Analysis

The virus strains from each positive pig farm were subjected to sequencing and phylogenetic tree analysis. The sequences have been uploaded to NCBI GenBank (PEDV S gene with GenBank accession numbers: OQ504178~OQ504184; PoRV VP4 gene with GenBank accession numbers: OQ504188~OQ504195; PoRV VP7 with GenBank accession numbers: OQ504197~OQ504204; PDCoV S gene with GenBank accession number: OQ504187).

**Figure 4:**
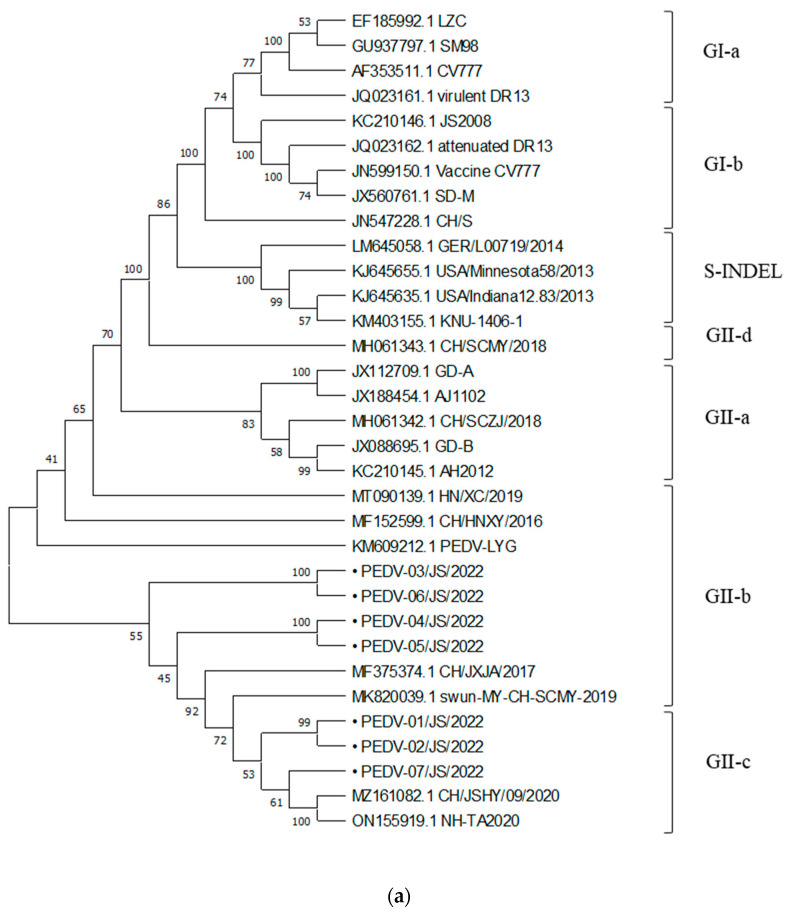

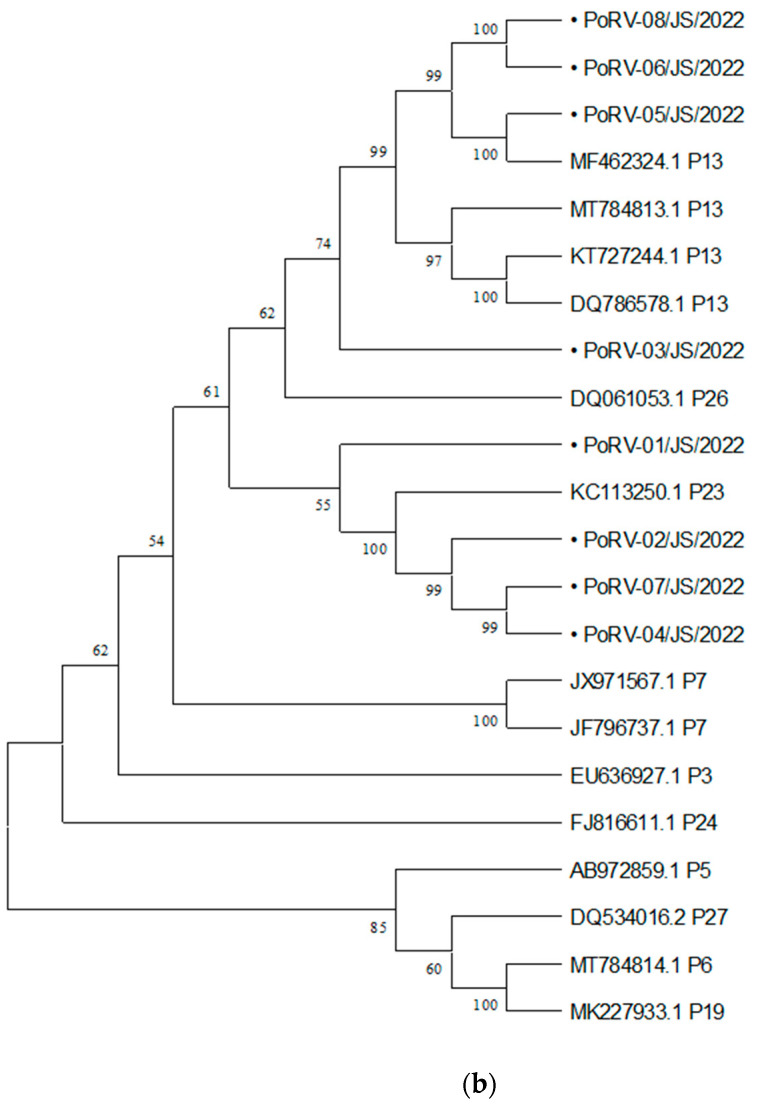

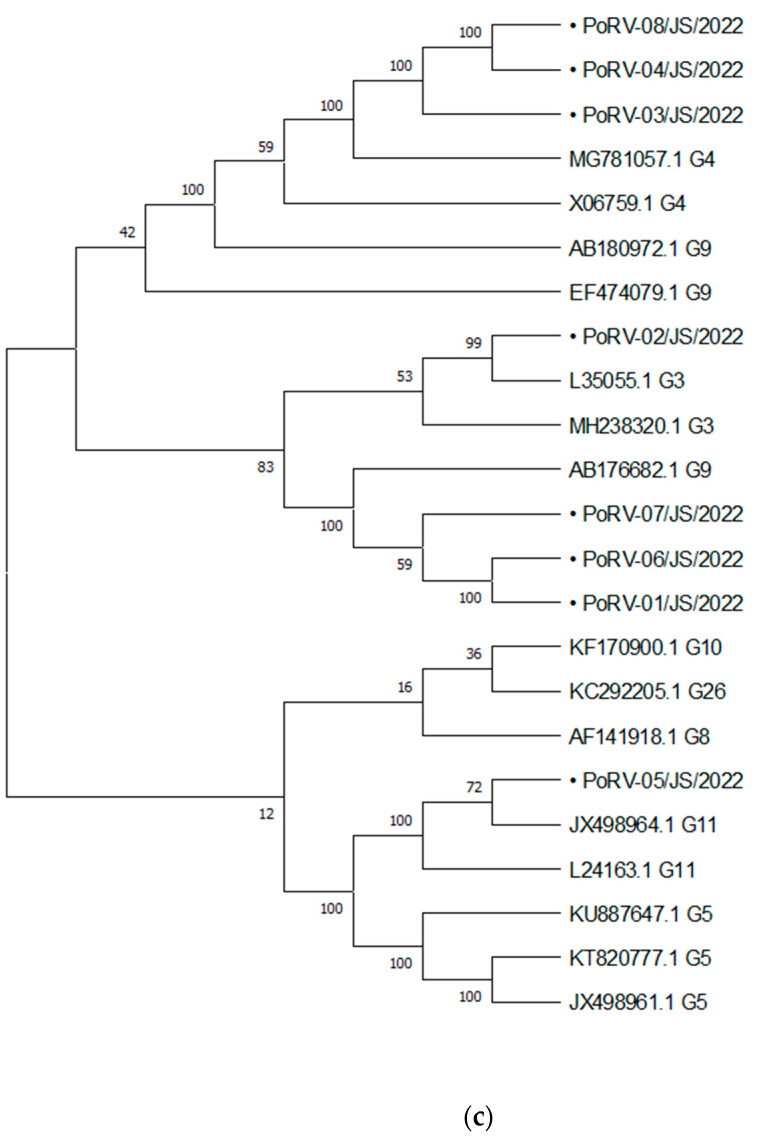

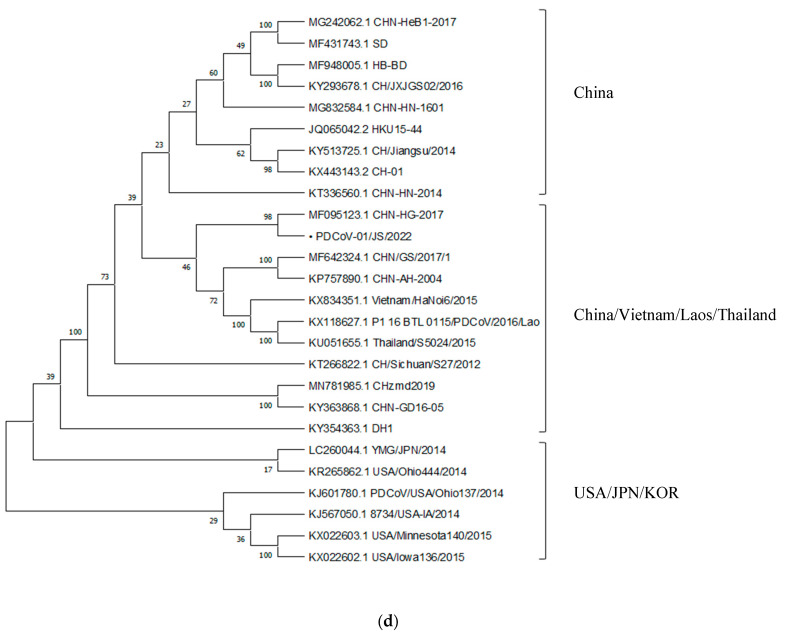
Phylogenetic tree based on the nucleotide sequence of the PEDV S gene (**a**), PoRV VP4 (**b**), PoRV VP7 gene (**c**), and PDCoV S gene (**d**), respectively. “•” indicates the positive samples tested in this study.

The results of phylogenetic analysis based on the PEDV S gene showed that four PEDV-positive samples detected in this study belonged to the GII-b subtype and three samples belonged to the GII-c subtype (Figure 4a). According to the phylogenetic tree based on the PoRV VP4 gene, four strains (PoRV-01, PoRV-02, PoRV-04, and PoRV-07) belonged to the P [23] type, and another four PoRV strains (PoRV-03, PoRV-05, PoRV-06, and PoRV-08) in this study belonged to the P [13] type (Figure 4b). The results of the VP7 gene phylogenetic analysis indicated that PoRV-03, PoRV-04, and PoRV-08 strains belonged to the G4 type, PoRV-02 belonged to the G3 type, PoRV-01, PoRV-06, and PoRV-07 belonged to the G9 type, and PoRV-05 belonged to the G11 type (Figure 4c). The detailed GP types of the eight PoRV-positive samples in this study are listed in Table 5. The results of phylogenetic analysis based on the PDCoV S gene showed that one PDCoV strain in this study belonged to the China/Vietnam/Laos/Thailand branch (Figure 4d).

## 4. Discussion

Porcine viral diarrhea is a common clinical disease that can lead to a high mortality rate in piglets. PEDV, PoRV, and PDCoV are important diarrhea viruses in piglets. On account of similar clinical symptoms and co-infections among them, it presents challenges to differentiate these three important porcine diarrhea viruses in clinical practice. Since 2010, highly pathogenic strains of PEDV have appeared and spread in many countries, causing severe economic losses in the global swine industry [24]. At the same time, millions of piglets died in more than 10 provinces in southern China due to PEDV variant strains [25]. In 2014, PDCoV spread to most states in the US, which also had a huge economic impact on the local pig industry [13]. In recent years, the infection rate of PoRV has constantly increased, and the co-infection between PoRV and PEDV or PDCoV has become common. From the perspective of controlling epidemic diseases, the first step is the rapid and accurate detection of etiological pathogens. Accurate identification of pathogens and fast differential diagnosis are crucial for controlling epidemics. Therefore, it is necessary to establish a detection method to distinguish PEDV, PoRV, and PDCoV simultaneously.

Several conventional RT-PCR assays or single-plex real-time RT-PCR have been developed for the detection of PEDV, PoRV, and PDCoV [26,27]. These methods are inefficient, time-consuming, and expensive compared to multiplex RT-PCR. Therefore, we developed a triplex real-time RT-PCR detection method for PEDV, PoRV, and PDCoV differentiation in this study. The triplex real-time RT-PCR assay established in this study had satisfactory specificity, sensitivity, repeatability, and reproducibility. To evaluate the performance of the established triplex real-time RT-PCR assay, 16 clinical samples of diarrheal piglets were used to compare the results between the triplex real-time RT-PCR assay and commercial single-plex real-time RT-PCR. The results of two detection methods were confirmed to be completely consistent, which suggested the triplex real-time RT-PCR assay in this study could replace the commercial single-plex real-time RT-PCR with high efficiency to differentiate PEDV, PoRV, and PDCoV simultaneously.

Next, 112 clinical samples were detected by triplex real-time RT-PCR to investigate the prevalence of PEDV, PoRV, and PDCoV in Jiangsu province, China. The results showed that 51.79% (58/112) were PEDV positive, 59.82% (67/112) were PoRV positive, and 2.68% (3/112) were PDCoV positive, respectively. From the above results, we could conclude that PEDV and PoRV are the major porcine diarrhea pathogens in Jiangsu province. Guangming Ding et al. developed a multiplex RT-PCR for PEDV, TGEV, PoRV, porcine kobuvirus (PKV), porcine sapovirus (PsAV), and PDCoV, and the positive rates of PEDV, PoRV, and PDCoV were 19.69% (78/398), 17.59% (70/398), and 36.18% (144/398) in 398 samples collected from North, Middle, and South China between 2015 and 2017 [28]. Shuo Jia et al. established a dual priming oligonucleotide (DPO)-based real-time RT-PCR assay for PEDV, TGEV, PoRV, and PDCoV and detected 672 diarrhea samples collected in Northeast China from 2017 to 2018, and the results showed 19.05% (128/672), 4.32% (29/672), and 3.87% (26/672) positive rates for PEDV, PoRV, and PDCoV, respectively [1]. The disparity in PEDV, PRoV, and PDCoV positive rates in these studies could be attributed to differences in sample collection time and geographical distribution of sampling.

In addition, the co-infections of porcine diarrhea viruses cannot be ignored, since some co-infection status could even cause more severe symptoms. Honglei Zhang et al. conducted a prevalence analysis of PDCoV in Henan pigs. They found that co-infection between PEDV and PDCoV was approximately 60%, and the co-infection enhanced the severity of diarrhea and worsened the disease in piglets [19,29]. The co-infection of PEDV and PoRV was also supposed to be the most severe form of piglet diarrhea, and the detection rate could reach 16–25% in Northeast China. In some provinces of China, the co-infection of PEDV and PoRV even became the main reason for piglet diarrhea [30]. In our study, PoRV and PEDV co-infections accounted for 38.81% of all PoRV positive cases (26/67), PoRV and PDCoV co-infections accounted for 2.99% of all PoRV positive cases (2/67), and no PEDV and PDCoV co-infection cases were found.

To further characterize the epidemic strains of porcine diarrhea viruses in Jiangsu province, seven PEDV strains, eight PoRV strains, and one PDCoV strain were selected to construct a phylogenetic analysis. The phylogenetic tree based on the PEDV S gene could be divided into three genotypes: GI, GII, and S-INDEL. The GI type included classical strains such as CV777 and SD-M; the GII type was mainly prevalent in PEDV strains in recent years. The phylogenetic tree based on the PEDV S gene showed that four PEDV strains in this study belonged to the GII-b subtype and three strains belonged to the GII-c subtype, which were closely related to the prevalent PEDV strains in China in recent years but far from the classic PEDV strains and PEDV strains abroad. This indicated that there was a possibility of inter-provincial transmission of PEDV epidemic strains in Jiangsu province, and these strains underwent significant genomic variation compared to classic strains, which may lead to the unsatisfactory protective effect of commonly used vaccines in pig farms.

The phylogenetic tree based on the PoRV VP4 gene and the PoRV VP7 gene indicated that eight PoRV strains in this study had six different combinations: G3 [P23] type, G4 [P13] type, G4 [P23] type, G9 [P13] type, G9 [P23] type, and G11 [P13] type, respectively. According to previous studies, the G5P [7] type was the most widely prevalent combination type globally [31]. However, with the continuous variation and recombination in PoRV genomes, different types of virus strains have emerged in China in recent years. In this study, new combination strains of G3 [P23] type, G4 [P23] type, and G9 [P13] type have emerged, indicating that the combination types of PoRV strains in Jiangsu province are plentiful, the virus is undergoing recombination, and it has unique genetic evolution characteristics.

The phylogenetic tree based on the PDCoV S gene in this study indicated that the reference strains can be divided into three groups. The USA/JPN/KOR group was mainly composed of strains from countries such as the United States of America, Japan, and South Korea. The China group was mainly composed of Chinese strains, and the China/Vietnam/Laos/Thailand group was mainly composed of strains from Southeast Asian countries such as China, Vietnam, Laos, and Thailand. The PDCoV-01 strain in this study belonged to the China/Vietnam/Laos/Thailand groups. This may be related to the frequent animal and animal product transactions and close personnel communication between China and Southeast Asian countries in recent years.

In conclusion, we developed a triplex real-time RT-PCR assay in this study to differentiate PEDV, PoRV, and PDCoV, which showed satisfactory specificity, sensitivity, repeatability, and reproducibility. The established triplex real-time RT-PCR can be well applied to the detection of clinical samples, indicating that the assay we have established in this study is a useful detection tool for rapid differential detection of PEDV, PoRV, and PDCoV.

## Figures and Tables

**Table 1:** Primers and probes used in this study. F, R, and P indicate forward primer, reverse primer, and probe, respectively.

Viruses	Primers/Probes	Sequences (5′–3′)	Reference
PEDV	PEDV-M-F	CACTCCTTAGTGGTACATTGCTTGTAGA	This study
	PEDV-M-R	CCTTGGCGACTGTGACGAA	This study
	PEDV-M-P	FAM-ACAGGTAAGTCAATTACC-MGB	This study
PoRV	PoRV-NSP5-F	GAAGTCTCCAGAGGATATTGGACC	This study
	PoRV-NSP5-R	TCTTAACTGCATTCGATCTAATCGA	This study
	PoRV-NSP5-P	ROX-CTGATTCTGCTTCAAACG-MGB	This study
PDCoV	PDCoV-N-F	CCTACTACTGACGCGTCTTGGTT	Adapted from [20]
	PDCoV-N-R	TGCCACGAAACTGAGGATGA	Adapted from [20]
	PDCoV-N-P	TAMRA-TGCTCAAAGCTCAAAAC-MGB	Adapted from [20]

**Table 2:** Intra-repeatability and inter-reproducibility of the triplex real-time RT-PCR assay.

Standard Sample	Target	Intra-Reproductivity (Ct Value)	SD	Coefficients of Variation (%)	Inter-Reproductivity (Ct Value)	SD	Coefficients of Variation (%)
6.0 × 10^6^ copies/μL	PEDV	14.15	0.36	2.56	14.35	0.49	3.41
	PoRV	15.08	0.05	0.35	15.13	0.11	0.74
	PDCoV	13.40	0.34	2.52	13.46	0.30	2.24
6.0 × 10^4^ copies/μL	PEDV	20.18	0.52	2.60	20.24	0.44	2.19
	PoRV	20.99	0.10	0.48	21.08	0.20	0.93
	PDCoV	20.99	0.59	2.66	20.73	0.68	3.32
6.0 × 10^2^ copies/μL	PEDV	26.00	1.12	4.32	25.23	1.02	4.04
	PoRV	27.83	1.22	4.39	27.71	1.04	3.75
	PDCoV	25.40	1.21	4.76	23.94	1.03	4.34

**Table 3:** The detection results of 16 piglet diarrhea samples using the commercial single-plex real-time RT-PCR detection kit and the triplex real-time RT-PCR assay in this study.

Sample	Commercial Real-Time RT-PCR Detection Kit			Triplex Real-Time RT-PCR Assay		
	PEDV	PoRV	PDCoV	PEDV	PoRV	PDCoV
1	+	-	-	+	-	-
2	+	-	-	+	-	-
3	+	+	-	+	+	-
4	+	+	-	+	+	-
5	+	-	-	+	-	-
6	+	+	-	+	+	-
7	+	+	-	+	+	-
8	+	+	-	+	+	-
9	-	+	-	-	+	-
10	+	+	-	+	+	-
11	+	-	-	+	-	-
12	-	+	-	-	+	-
13	-	+	-	-	+	-
14	-	+	-	-	+	-
15	-	+	-	-	+	-
16	-	-	+	-	-	+

**Table 4:** The detection results of 112 clinical diarrhea samples.

PEDV+	PoRV+	PDCoV+	PEDV+PoRV+	PDCoV+PoRV+
58/112	67/112	3/112	26/112	2/112

**Table 5:** GP type of eight PoRV-positive samples in this study.

No.	Strain Name	G[P] Type
1	PoRV-01	G9 [P23]
2	PoRV-02	G3 [P23]
3	PoRV-03	G4 [P13]
4	PoRV-04	G4 [P23]
5	PoRV-05	G11 [P13]
6	PoRV-06	G9 [P13]
7	PoRV-07	G9 [P23]
8	PoRV-08	G4 [P13]

## Data Availability

All the data generated during the current study are included in the manuscript. Additional data related to this article may be requested from the corresponding authors.

## Supplementary Materials

The following supporting information can be downloaded at: https://www.mdpi.com/article/10.3390/v15061238/s1, Table S1: Specific information about the detection results of 112 clinical diarrhea samples.
